# Auricular Ultrasonic Vagus Nerve Stimulation: Effectiveness of Blinding and Occurrence of Adverse Effects in Healthy Volunteers

**DOI:** 10.3390/brainsci15090986

**Published:** 2025-09-13

**Authors:** Bas Labree, Marcus Kaiser, Mohamad A. Pourhoseingholi, Derek J. Hoare, Magdalena Sereda

**Affiliations:** 1 NIHR Nottingham Biomedical Research Centre, Nottingham NG1 5DU, UK; bas.labree1@nottingham.ac.uk (B.L.); marcus.kaiser@nottingham.ac.uk (M.K.); amin.pourhoseingholi@nottingham.ac.uk (M.A.P.); derek.hoare@nottingham.ac.uk (D.J.H.); 2 Hearing Sciences, Mental Health and Clinical Neurosciences, School of Medicine, University of Nottingham, Nottingham NG7 2RD, UK; 3 Precision Imaging, Mental Health and Clinical Neurosciences, School of Medicine, University of Nottingham, Nottingham NG7 2UH, UK; 4 Department of Neurosurgery, Ruijin Hospital, Shanghai Jiao Tong University School of Medicine, Shanghai 200025, China; 5 Department of Speech and Hearing Sciences, University College Cork, T12 EK59 Cork, Ireland

**Keywords:** ultrasound stimulation, vagus nerve, blinding, adverse effects

## Abstract

Background/Objectives: Both invasive and non-invasive electrical stimulation of the vagus nerve have been studied as potential treatments for neurological conditions, with mixed results. Ultrasonic Vagus Nerve Stimulation (U-VNS), which non-invasively stimulates the auricular branch of the vagus nerve using ultrasound, may offer a more targeted and effective approach than electric currents. To facilitate future clinical trials of U-VNS, this study aimed to (1) investigate the effectiveness of blinding of a U-VNS device versus a sham device and (2) record the type, onset, and duration of any adverse effects resulting from U-VNS. Methods: In this single-blind randomised controlled study, twenty healthy volunteers were randomly assigned to receive either a 30 min session of true U-VNS and a 30 min session of sham stimulation 1 week later, or vice versa. The effectiveness of blinding and the occurrence of adverse effects were measured using self-report questionnaires. Results: James’ Blinding Index showed that blinding using the sham device was highly effective in both the real U-VNS condition, BI = 0.9 (95% CI: 0.7–1.0), and the sham condition, BI = 1.0 (95% CI: 1.0–1.0). The adverse effects reported were mild, transient, and mostly related to sensations on the skin immediately under the transducer of the device. Conclusions: A high level of blinding effectiveness can be achieved for U-VNS by using a sham device. Adverse effects are generally mild and transient. These findings will inform the design of future clinical trials of U-VNS.

## 1. Introduction

The vagus nerve (cranial nerve X) is a large nerve that runs from the gut, through the abdomen and chest and connects to the brain stem at the medulla oblongata. It consists of two main sections, left and right, and comprises a series of branches. One of these, the auricular branch, innervates the ear [1]. External stimulation of the vagus nerve, so-called Vagus Nerve Stimulation (VNS), has been used to treat a number of health conditions associated with the functioning of the vagus nerve [2].

Given the anatomy of the vagus nerve, several different approaches to the delivery of this stimulation have been attempted. These can be divided into two categories: invasive and non-invasive. Invasive VNS employs an electrode wrapped around a section of the vagus nerve in the neck, connected via a lead to a processor implanted in the chest [2]. Due the invasive nature of this approach and the possibility of surgical complications, non-invasive alternatives have been explored. Non-invasive VNS aims to stimulate one of the branches of the vagus nerve that are accessible through the skin. One candidate stimulation site is the auricular branch of the vagus nerve, as it can be accessed via a stimulator placed on the ear [3]. Stimulation of this section of the vagus nerve is believed to lead to activation of the locus coeruleus and nucleus of the solitary tract, two areas involved in the regulation of excitatory-inhibitory balance, i.e., via arousal and attention [4]. Indeed, non-invasive electrical VNS has been trialled for a range of disorders including anxiety [5], epilepsy [6], tinnitus [7,8], chronic pain [9], and migraine [10]. Current evidence on the efficacy of electrical VNS on tinnitus is limited due to the small sample sizes, high risk of bias and limited quality of reporting. However, a recent systematic review found that most studies of electrical VNS for tinnitus report a small decrease in tinnitus symptom severity or distress [7]. There is no consensus on why these reported effects are small, but it may relate to how electric currents move through organic tissue. As electric currents follow the path of least resistance, a current applied to the skin may dissipate into a diffuse electric field, with only a limited amount of current reaching the intended stimulation target, in this case the vagus nerve.

Focused Ultrasound Stimulation (FUS), which uses ultrasound waves rather than electric currents, has recently been introduced as an alternative to electrical neuromodulation, offering greater spatial resolution, among other advantages [11,12]. Importantly, electric currents follow the path of least resistance and electrical stimulation may result in a relatively diffuse electric field which may or may not include the target site at sufficient density to elicit neurophysiological changes. Ultrasound stimulation, by contrast, has greater spatial focus, and has been shown to be able to induce neuronal activity, for instance by eliciting phosphenes and muscle contractions [13]. The principle of substituting ultrasound waves for electric currents can also be applied to VNS in the form of Ultrasonic Vagus Nerve Stimulation (U-VNS). To this end, the ZenBud device was developed by NeurGear Inc. This device delivers ultrasound stimulation to the auricular branch of the vagus nerve via a transducer mounted in a headset. Developed as a device to combat stress and induce relaxation, its effects on symptoms of anxiety disorders have recently been studied, with 85% of participants reporting improvements [14].

If potential future clinical applications of U-VNS are to be informed by clinical trials, a blinded sham condition is necessary. Furthermore, whilst extensive safety testing has taken place and the ZenBud has received a CE mark, a detailed understanding of any mild adverse effects associated with U-VNS is necessary to ensure participants in such future trials are fully informed when consenting to trials. This study aimed to (1) investigate the effectiveness of blinding of a U-VNS device versus sham control, and (2) record the type, onset, and duration of any adverse effects resulting from U-VNS.

## 2. Materials and Methods

This study received favourable opinion from the Faculty of Medicine and Health Sciences Ethics Committee of the University of Nottingham (ethics reference number FMHS 194-0524).

### 2.1. Participants

A sample size calculation was performed in accordance with established practice for crossover designs [15]:

n = ((Z_{1−α/2} + Z_{1−β})^2 × 2 × (1 − ρ))/d^2

For a significance level (α) of 0.05 and to achieve 80% power (1 − β = 0.8), assuming a medium to large effect size (Cohen’s d = 0.7), based on previous work [16] and a moderate correlation between the conditions (ρ = 0.5), a sample size of 16 would be required. However, to account for attrition, 20 healthy volunteers were recruited via word of mouth, departmental email lists, posters, and adverts distributed at the University of Nottingham and placed online. Potential participants were considered eligible if they were aged 18 or over, in good general health, not taking any medication (excluding the contraceptive pill) at the time of recruitment, and were able and willing to remove any ear piercings. Potential participants were excluded if they had a current or past diagnosis of any neurological or psychiatric conditions, current or past experience of cardiac arrhythmia, had used medication or recreational drugs that affect the nervous system in the previous 3 months, were pregnant, or had an allergy to ultrasound gel or any of its components (propylene glycol, glycerin, isothiazolinones).

### 2.2. Intervention and Comparator

**Figure 1:**
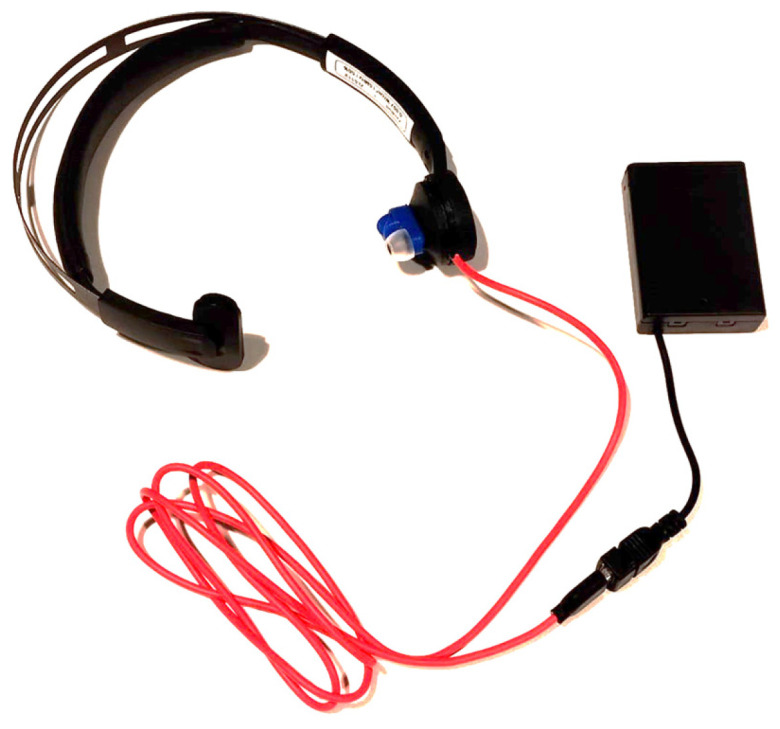
The ZenBud device.

U-VNS was delivered using the ZenBud device manufactured by NeurGear (Rochester, NY, USA), specifically designed to deliver ultrasound stimulation to the auricular branch of the vagus nerve. The ZenBud device used for this trial (NeurGear, Rochester, NY, USA; Figure 1) is a CE-compliant over-the ear headset. It delivers low-intensity focused ultrasound to the auricular branch of the vagus nerve through several layers of skin (centre frequency 5.3 MHz, pulse repetition rate 41 hertz, 50% duty cycle, average intensity of 1.03 MPa). As a safety measure, the device shuts down automatically after running for 29 min, which limits the duration of use [14]. Unlike the standard version of the ZenBud, this device was custom made to stimulate the left auricular branch instead of the right, to align with previous data on electrical VNS. The wearer may experience a sensation of warmth on the skin where the transducer sits. The device also emits a sound whilst stimulating, to indicate it is switched on.

The sham protocol was delivered using a sham device, also produced by NeurGear, which is identical in appearance to the true ZenBud device, emits the same sound, and warms up slightly where the transducer sits on the skin. Being a sham version of the standard ZenBud, the (non-functional) transducer sat on the right ear.

### 2.3. Procedure

Participants attended two appointments, 1 week apart. Ten participants were randomly assigned to receive either true U-VNS at the first session and sham at the second session, and the other ten to have sham at the first session and true U-VNS at the second. The randomisation was completed electronically by a research assistant with no other involvement in this study. Only participants were blinded. At the first session, informed consent was obtained and eligibility confirmed using a safety screening questionnaire. At the second appointment, eligibility was confirmed verbally. Following this, participants sat in a quiet room with an investigator whilst undergoing stimulation (or sham) using the ZenBud device for 29 min. At the end of each session, participants completed an in-house questionnaire to assess the effectiveness of blinding and adverse effects. At the end of the second session, after all data had been obtained, participants were unblinded verbally.

### 2.4. Data Analysis

With regard to effectiveness of blinding, participants were asked at the end of each session “Do you believe you received real or sham stimulation?” and given the options *Real*, *Sham*, and *I don’t know* to choose from. The responses were used to calculate James’ Blinding Index [15]:

BI = [1 + *Ρ*_DK_ + (1 − *Ρ*_DK_) *Κ*_D_]/2, where *Κ*_D =_ (*Ρ*_Do_ − *Ρ*_De_)/*Ρ*_De_

With regard to adverse effects, participants were asked at the end of each session to “Please indicate the extent to which, if any, you felt the following sensations by using the scale below”, followed by a list of previously reported adverse effects associated with tDCS: *Itching*, *Burning*, *Pain*, *Tingling*, *Headache*, *Warmth/heat*, *Metallic taste*, *Fatigue*, *Nausea*, *Redness*, *Other* and the rating options *None*, *Mild*, *Moderate*, and *Severe*. Finally, participants were asked “How long did the sensations last?”, with the options *It stopped quickly*, *It stopped around the middle of the session*, *It stopped around the end of the session*, and *It continued after the end of the session*.

## 3. Results

### 3.1. Demographic Information

Of the 20 participants, 14 (70%) were female, 5 (25%) were male, and 1 (5%) was other sex. Participants were aged between 18 and 62 years (mean = 39.1, standard deviation = 12.1). There was no loss to follow-up.

### 3.2. Blinding

James’ Blinding Index (BI) was calculated to assess the effectiveness of blinding. For the real intervention, BI = 0.9 (95% CI: 0.7–1.0), for sham, BI = 1.0 (95% CI: 1.0–1.0), indicating a high level of blinding effectiveness (Table 1).

### 3.3. Adverse Effects

Adverse effects reported by participants were generally uncommon, mild, and transient. Most related to sensations on the skin where the transducer rested on the ear. Only four participants reported adverse effects that persisted past the stimulation session. All did so following both true U-VNS and sham. One reported sensations lasting 5–10 min after stimulation; one described them ending “shortly and another “briefly” after. One participant reported painful skin persisting for 30 min after the end of the session. Table 2 and Table 3 provide a full overview of all adverse effects reported in each condition.

## 4. Discussion

This study aimed to (1) investigate the effectiveness of blinding of a U-VNS device versus a sham control and (2) record the type, onset, and duration of any adverse effects resulting from U-VNS. This study was the first to evaluate the effectiveness of blinding and adverse effects of this device. It was also the first to apply the maximum duration of 29 min.

The level of blinding effectiveness achieved ranged from high in the real stimulation condition to perfect in the sham condition. This level of blinding is promising for future trials of the ZenBud device. However, it should be noted that investigator blinding was not possible in this study, but would ideally be applied to future studies of this device.

Very few adverse effects were reported. Those that were tended to be mild and transient, with some notable exceptions. Most adverse effects related to sensations on the skin, most often described as tingling, burning, or pain. Participants frequently attributed these effects not to the U-VNS, but to the pressure of the transducer on the skin. This was the case, for instance, for the two reports of “moderate pain” in each condition. A few non-skin-related adverse effects were reported, most notably sound discomfort, caused by the sound emitted by the ZenBud device. Finally, one participant reported numbness and coldness of the hand, which they reported as severe in the sham condition and moderate in the real U-VNS condition. Given this difference in reported severity, and in the absence of an obvious mechanism for this sensation to be induced by the intervention, this experience may have been psychosomatic.

In conclusion, future clinical trials of the efficacy and/or safety of the ZenBud device for conditions involving the vagus nerve may rely on the use of a ZenBud sham device as a highly blindable placebo control. The findings on adverse effects reported here should be used to inform prospective participants in such trials with regard to what adverse effects they may expect.

## Figures and Tables

**Table 1:** Results of the blinding questionnaire.

	N	Real	Sham	I Don’t Know
Real U-VNS	20	6	2	12
Sham U-VNS	20	6	4	10

**Table 2:** Results of the adverse effects questionnaire after real U-VNS.

	None	Mild	Moderate	Severe
Itching	18	1	1	0
Burning	18	2	0	0
Pain	11	7	2	0
Tingling	17	3	0	0
Headache	17	3	0	0
Warmth/heat	16	4	0	0
Metallic taste	0	0	0	0
Fatigue	19	1	0	0
Nausea	0	0	0	0
Redness	11	9	0	0
Other *	14	3	2	1
**Total**	**141**	**33**	**5**	**1**

* Other adverse effects reported: mild discomfort, moderate discomfort, severe loudness discomfort, mild noise in ear, mild acoustic disturbance, and moderate numbness (hand).

**Table 3:** Results of the adverse effects questionnaire after sham U-VNS.

	None	Mild	Moderate	Severe
Itching	20	0	0	0
Burning	18	1	1	0
Pain	14	4	2	0
Tingling	11	8	1	0
Headache	19	1	0	0
Warmth/heat	14	5	1	0
Metallic taste	0	0	0	0
Fatigue	19	0	1	0
Nausea	0	0	0	0
Redness	10	10	0	0
Other *	0	4	0	2
**Total**	**125**	**33**	**6**	**2**

* Other adverse effects reported: mild ache, mild pressure, mild mental fatigue from sound, mild ear pain, severe numbness (hand), and severe coldness (hand).

## Data Availability

All data for which consent to share has been obtained will be shared via the University of Nottingham data archive under a CC-BY licence.

## References

[1] Câmara R., Griessenauer C.J., Tubbs R.S., Rizk E., Shoja M.M., Loukas M., Barbaro N., Spinner R.J. (2015). Chapter 27—Anatomy of the Vagus Nerve. Nerves and Nerve Injuries.

[2] Johnson R.L., Wilson C.G. (2018). A review of vagus nerve stimulation as a therapeutic intervention. J. Inflamm. Res..

[3] Clancy J.A., Mary D.A., Witte K.K., Greenwood J.P., Deuchars S.A., Deuchars J. (2014). Non-invasive vagus nerve stimulation in healthy humans reduces sympathetic nerve activity. Brain Stimul..

[4] Shiozawa P., Silva M.E.d., Carvalho T.C.d., Cordeiro Q., Brunoni A.R., Fregni F. (2014). Transcutaneous vagus and trigeminal nerve stimulation for neuropsychiatric disorders: A systematic review. Arq. Neuro-Psiquiatr..

[5] Groves D.A., Brown V.J. (2005). Vagal nerve stimulation: A review of its applications and potential mechanisms that mediate its clinical effects. Neurosci. Biobehav. Rev..

[6] Englot D.J., Chang E.F., Auguste K.I. (2011). Vagus nerve stimulation for epilepsy: A meta-analysis of efficacy and predictors of response: A review. J. Neurosurg..

[7] Stegeman I., Velde H., Robe P., Stokroos R., Smit A. (2021). Tinnitus treatment by vagus nerve stimulation: A systematic review. PLoS ONE.

[8] Hoare D.J., Shorter G.W., Shekhawat G.S., El Refaie A., Labree B., Sereda M. (2024). Neuromodulation treatments targeting pathological synchrony for tinnitus in adults: A systematic review. Brain Sci..

[9] Chakravarthy K., Chaudhry H., Williams K., Christo P.J. (2015). Review of the uses of vagal nerve stimulation in chronic pain management. Curr. Pain Headache Rep..

[10] Song D., Li P., Wang Y., Cao J. (2023). Noninvasive vagus nerve stimulation for migraine: A systematic review and meta-analysis of randomized controlled trials. Front. Neurol..

[11] Kim S., Jo Y., Kook G., Pasquinelli C., Kim H., Kim K., Hoe H.-S., Choe Y., Rhim H., Thielscher A. (2021). Transcranial focused ultrasound stimulation with high spatial resolution. Brain Stimul..

[12] Rezayat E., Toostani I.G. (2016). A review on brain stimulation using low intensity focused ultrasound. Basic Clin. Neurosci..

[13] Blackmore J., Shrivastava S., Sallet J., Butler C.R., Cleveland R.O. (2019). Ultrasound neuromodulation: A review of results, mechanisms and safety. Ultrasound Med. Biol..

[14] Kohler I., Hacker J., Martin E. (2025). Reduction of Anxiety-Related Symptoms Using Low-Intensity Ultrasound Neuromodulation on the Auricular Branch of the Vagus Nerve: Preliminary Study. JMIR Neurotechnol..

[15] James K.E., Bloch D.A., Lee K.K., Kraemer H.C., Fuller R.K. (1996). An index for assessing blindness in a multi-centre clinical trial: Disulfiram for alcohol cessation—A VA cooperative study. Stat. Med..

[16] Deng A., Adams B., Scutt P., Hoare D.J., Sereda M. (2023). Effectiveness of Blinding and Occurrence of Adverse Effects in Sham-Controlled Transcranial Direct Current Stimulation (tDCS) Study.

